# Words-Deeds Gap for the Purchase of Fairtrade Products: A Systematic Literature Review

**DOI:** 10.3389/fpsyg.2019.02705

**Published:** 2019-12-03

**Authors:** Elena Kossmann, Monica Gomez-Suarez

**Affiliations:** Department of Finance and Marketing Research, Administration and Business Faculty, Universidad Autónoma de Madrid, Madrid, Spain

**Keywords:** fair trade, systematic review, ethical purchasing gap, consumer behavior, words-deeds gap, ethical consumerism, values, theory of planned behavior

## Abstract

**Rationale:** Nowadays ethical consumerism is part of the agenda of consumers, businesses, and governments for its promise of a brighter future through the consumption of goods with added social or environmental value. Consumption of fair trade goods has been enjoying huge growth in the last few years as people are becoming more conscious of their consumption practices.

**Objective:** In this piece of research we identify existing literature on the words-deeds gap for the purchase of fairly traded products. Moreover, we present and analyze the moderators to the ethical purchasing gap.

**Method:** A collection of research articles was compiled using a key-word search in 3 databases: Scopus, Springerlink, and Sciencedirect. The research was carried out using various groups of keywords and completed using the following three criteria: articles published in English, between 2010 and 2018, in refereed journals. Further to the systematic literature review, all pertinent articles were imported into Atlas.ti for further thematic analysis.

**Results:** Two thousand and seventy eight articles were identified from which 32 met the inclusion criteria. The content analysis resulted in the following themes: Values, Theory of Planned Behavior and Theory of Reasoned Action, Willingness-to-pay, Labels and Certification, Religion, Guilt, Dual Attitudes, Demographics.

**Conclusion:** Despite “moral” products tasting better and self-claims to this direction, there is still a gap between claimed and actual consumption. Research has mostly concentrated on different perspectives, such as willingness to pay, items from the theory of planned behavior or theory of reasoned action. Given the recent developments in the theory of dual attitudes and further to our research this area is currently underrepresented in FT research and could provide useful insights that may increase consumption of FT products.

## Introduction

The ever-increasing private consumption levels in developed and newly industrialized countries are negatively impacting society and the environment at large (Pereira Heath and Chatzidakis, 2012; Barbarossa and De Pelsmacker, 2016). Slave-like working conditions for the weakest on the supply chains, climate change, and destruction of natural habitats are but a few of the issues humanity is faced with. Achieving a sustainable future requires that people change their consumption habits (McKenzie-Mohr and Schultz, 2014, p. 35) with the aim to change unsustainable production patters. The papers presented in this systematic literature review have identified factors that affect or contribute to the consumer decision-making for fair trade products, fair trade being part of an overall sustainability agenda.

In 2016, 815 million people or one in nine people on earth went to bed with an empty stomach (World Food Programme, 2018). In just 1 year this number increased by 38 million (FAO et al., 2017). At the same time, the gap between the rich and the poor keeps widening. In many countries, it is at its highest in 30 years (OECD, 2015, pp. 3, 15). Shockingly, 85% of the world's poor live in rural areas (Alkire et al., 2014). Ironically, many of those who produce our food we eat or the clothes we wear find it hard to feed themselves or their families. Moreover, income inequality does not only have social and political implications but is also known to inhibit GDP growth (OECD, 2015, p. 3), due to decreased access to quality education, which in turn implies “large amounts of wasted potential and lower social mobility” (OECD, 2015, pp. 3, 15). Due to income inequality trust in institutions falls, while the social fabric frays.

Ethical consumption defined as “the purchase of a product that concerns a certain ethical issue and that a consumer chooses freely” (Doane, 2001, p. 6), has grown considerably in terms of market value in the last few years (Carrigan and De Pelsmacker, 2009; The Nielsen Company, 2014; Fairtrade Labelling Organizations International (FLO), 2016). Consumers decide to consume “ethically” as a response to eminent political or moral issues associated with traditional production and trade patterns. Despite the large number of consumers who identify themselves as ethical, the value of “ethical” products sold is much lower than the number of consumers who describe themselves as caring for sustainability issues (Carrington et al., 2010, 2016). This phenomenon is known as ethical purchase gap or words-deeds gap (Carrington et al., 2010, 2014). Consequently, numerous academics have researched decision-making behind ethical or sustainable products in order to elucidate the moderators of the ethical purchasing gap (De Pelsmacker et al., 2005; Chatzidakis et al., 2007, 2016; Newholm and Shaw, 2007; Carrington et al., 2010, 2014; Barbarossa and De Pelsmacker, 2016; Hassan et al., 2016). Despite the increased academic interest in the question of the words-deeds gap, to the best of our knowledge this is the first systematic literature review with the aim to collect the results from the different pieces of research and present them in a comprehensive form.

Fairtrade (FT) and other organizations part of the fair trade movement, with a focus on the Global South, seek to remedy the effects of unregulated trade by working to guarantee that the minimum price paid mainly for agricultural, as well as other produce, covers the minimum costs of production, but also through the FT Premium, communities are provided with a sum of money that they are free to invest in their communities in the best way they see fit, further to democratic processes (Fairtrade Labelling Organizations International (FLO), 2018). Despite the global economic crisis, FT sales have grown exponentially in the last few years (Fairtrade Labelling Organizations International (FLO), 2018; Fairtrade Global Sales Overview, n.d.). Similarly, the academic interest in Fair Trade has dramatically increased. Nevertheless no systematic literature review has been conducted to synthesize the results of the academic research on the words-deeds gap for FT products and to the best of our knowledge no paper has been published after 2009 (Carrigan and De Pelsmacker, 2009) that has attempted to understand Fairtrade consumer behavior during the crisis.

Consumers form attitudes through complex processes. Consumer behavior is influenced by attitudes, situational cues as well as other factors. The relationship between attitudes and behavior is complex (Solomon et al., 2006, p. 138). In this paper, we present the most recent contributions that highlight factors, which affect attitudes and behavior toward fair trade products. In the reviewed paper factors such as inter alias willingness to pay, neutralization, values, guilt, and pride have been identified to contribute to the complex phenomenon of ethical purchasing for fair trade products.

The proliferation of articles on attitude prove that it is a central focal point of theory and research (Ajzen, 2001). Attitude represents a “summary evaluation of a psychological object” (Ajzen, 2001, p. 28). It may have dimensions such as good/bad, pleasant/unpleasant etc. Traditional models of human behavior assumed a one-dimensional attitude to any single psychological object. Recent theories of dual attitudes however prove that an individual may have two distinct attitudes toward a given object. The new attitude may override but not replace the old one (Wilson et al., 2000, p. 101). The cognitive ability of an individual to retrieve one or the other attitude determines which attitude the individual exhibits.

The Theory of Reasoned Action (TRA) (Fishbein and Ajzen, 1975) and the Theory of Planned Behavior (TPB) (Ajzen, 1991) have highlighted the relationship between attitudes and behavior. These theories have been referenced in many pieces of research for different types of ethical purchasing (Hassan et al., 2016). TRA suggests that actual behavior is determined by behavioral intention. Behavioral intentions are formed based on subjective norms and attitudes toward the behavior. Subjective norms are defined as “the perceived social pressure to perform or not to perform the behavior” (Ajzen, 1991). They represent an individual's perception of how most people who matter to him would consider his actions (Trafimow et al., 2002). Research has proved that attitude predicts behavior to different extents. Some attitudes prove to be stronger than others and as such have a greater predictive power than others (Ajzen et al., 2009).

Despite the focus of researchers on this area, the words-deeds gap persists. It is for this reason and due to the potential of fair trade to offer solutions to many of the problems mentioned above, that we chose to conduct this systematic literature review.

This article presents a systematic literature review covering pertinent, English-language, peer-reviewed journal articles on the words-deeds gap for FT products. With this review we aim to provide a comprehensive analysis of all relevant published research between 2010 and 2018 to elucidate areas of research that are of relevance not only to academics, but also to practitioners. Therefore, its intended contribution is to comprehensively present the current status of research and thus provide the basis for further research on the words-deeds gap.

## Materials and Methods

### Search Strategy

Our research objective and the unique contribution of this article is to capture all possible outcome factors that contribute to the attitude-behavior or words-deeds gap and have been analyzed in the literature. Therefore, we used several combinations of keywords to ensure that no seminal contributions have been omitted in this search and thus minimize the risk of bias. We looked for specific contributions to implicit attitudes and nudges, as these are two areas of increased interest to practitioners (Kossmann and Gómez-Suárez, 2018). In the case of the seventh combination, we intentionally used the Boolean operator NEAR instead of AND, as we are looking for articles that are particularly related to FT purchases and not simply articles that mention both words.

The keywords used are:

1. “fairtrade” OR “fair trade” AND “shopper experience”
2. “fairtrade” OR “fair trade” AND “customer experience”
3. “fairtrade” OR “fair trade” AND “multi-channel”
4. “fairtrade” OR “fair trade” AND “consumer experience”
5. “fairtrade” OR “fair trade” AND “implicit attitudes”
6. “fairtrade” OR “fair trade” AND “nudge”
7. “fairtrade” OR “fair trade” NEAR “purchase.”

We then proceeded to the choice of databases for the keyword search. Firstly, we consulted CiteScore Metrics 2017 to derive a list of peer-reviewed marketing publications. We identified 142 journals under the Scopus sub-subject area Marketing (ASJC Code 1406). We then searched for suitable databases to access those journals and chose:

- Science Direct: includes 29 marketing journals.
- Scopus: includes 198 marketing journals according to its website it “indexes content from 24,600 active titles and 5,000 publishers which is rigorously vetted and selected by an independent review board, and uses a rich underlying metadata architecture to connect people, published ideas and institutions” (Elsevier, 2019).
- Springer Link: 19 marketing journals in English.

### Eligibility Criteria

All articles in the final list met the following criteria:

1. Year of publication: 2010–2018: we chose this time frame as we aim to present the state-of-the-art research in this area.
2. English-language publications.
3. Publications in peer-reviewed journals.

### Study Selection

**Figure 1:**
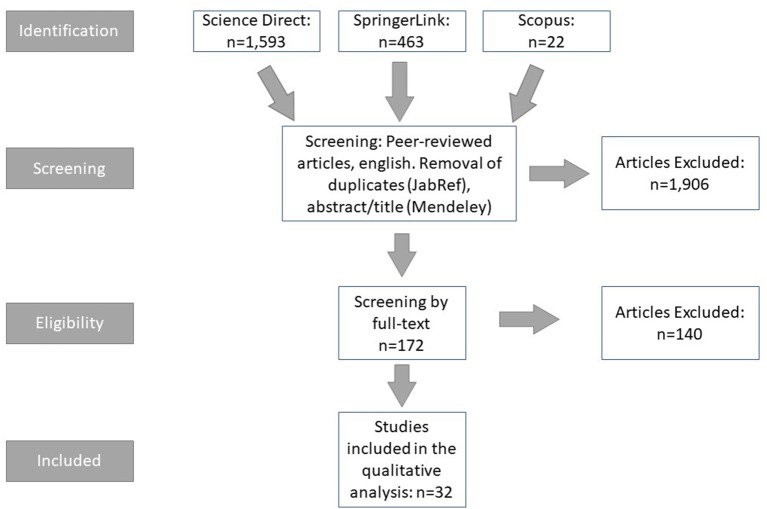
Study selection process.

Having applied the aforementioned eligibility criteria, we performed several searches using the keyword combinations mentioned above. After each keyword search, we downloaded all articles and saved them in several folders in Mendeley according to their source, keyword combination and date. Then, we exported all downloaded articles into (JabRef, n.d.), through which we in turn produced several Excel spreadsheets, one for each folder with the criteria mentioned above. In Excel we firstly cleaned for duplicates. Thereafter, we scanned all titles and if necessary, abstracts of the articles in order to derive the preliminary list of pertinent articles. We marked our decision on a separate column within the Excel spreadsheets. As mentioned previously key criterion in this step was whether the article answers the question of decision-making processes for Fairtrade products. This resulted in the preliminary list (see step “Eligibility”) with 172 articles. Finally, we read articles in the preliminary list in full to derive the final list. For the above process, we used the guidelines proposed in the PRISMA statement (Moher et al., 2015). The results of our search can be summarized in the following framework (Figure 1).

We firstly import all pertinent articles in Mendeley and run a first review attaching relevant tags. Followingly, we exported the final article list with their key attributes in Microsoft Excel to allow for the preliminary descriptive analysis below. Thereafter, we imported the articles to Atlas.ti in order to facilitate further analysis, including the thematic analysis. For this part of the analysis a two-level Code Book was developed beforehand and uploaded to Atlas.ti. We used Malhotra seminal handbook 2010 as basis for the classification of the methods used in the research.

Codes were grouped into the following categories (Table 1).

**Table 1:** Code groups.

**Code groups**
Causal research design
Conclusive research design
Continent
Country
Descriptive research
Exploratory research design
Observation
Observation by mode of administration
Qualitative research procedures
Quantitative data analysis
Research design
Research methodologies
Sample size
Sampling method
Secondary data
Survey
Syndicated sources: Household data
Theoretical approach
Year of publication

PICOS (Liberati et al., 2009) is an indispensable framework used in systematic reviews for clinical studies. PICOS has been utilized in this piece of research as part of PRISMA framework. PICOS stands for population or participants, interventions, comparators, outcomes and study design. Having carefully studied the articles resulting from our keyword search we concluded that strictly conforming to PICOS would not be suitable for marketing research. For example, not all articles report on interventions in a strictly clinical sense, as some articles capture only the status quo, and therefore there is no comparison of the state before and the state after an intervention or change in conditions. Therefore, we analyzed and categorized the articles according to their sampling technique (P or population), outcomes (O), and study design (S) (see Appendix B). All other aspects of PRISMA have been considered and provide the basis for this piece of research, with all individual steps clearly protocolled.

### Criteria for Inclusion and Exclusion

**Figure 2:**
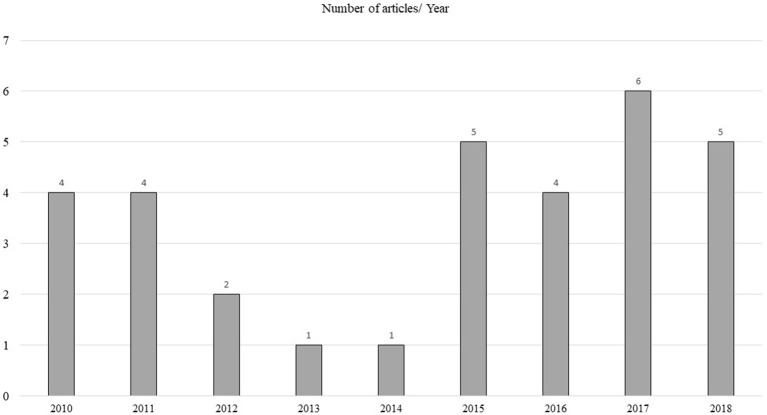
Number of articles published per year.

The results of the database research identified a total of 32 articles (Figure 2) for inclusion in the final list. After the removal of duplicates and keeping only the articles that were pertinent to the topic the number of articles dropped to 172. All articles in the preliminary list of pertinent articles were read to ensure that they offered insights into the words-deeds gap, in other words the attitudes-behavior or intention-behavior gap for FT products. Articles that did not specifically contribute to the further understand of the phenomenon of ethical purchasing gap were excluded from the final list. This part of the research took place between the 13th of November and 16th of December 2018. Both authors reviewed the 172 articles independently. The results of this review were uniform, so that no further steps needed to be taken.

## Results

### Studies' Characteristics

Table 2 shows the method of collecting the data. Three papers were theoretical and 29 empirical. 85% of the articles employed quantitative methods in the period 2010 to 2018, while only 2 employed mixed methods. The most used methods to collect information for the main study were experimental design (50%) and survey (40%). Twenty eight percentage used random samples. The rest employed non-probabilistic methods, mainly convenience samples.

**Table 2:** Number of articles per research methodology.

**Research method**	**Number of articles**	**Authors**
Empirical: Qualitative	27	Doran, 2010; Kim et al., 2010; Bondy and Talwar, 2011; Doran and Natale, 2011; Fennis et al., 2011; Langen, 2011; Kimura et al., 2012; Schuldt et al., 2012; Antonetti and Maklan, 2014; Bratanova et al., 2015; Ladhari and Tchetgna, 2015; Lee et al., 2015; Rousseau, 2015; Van Loo et al., 2015; Akaichi et al., 2016; Chatzidakis et al., 2016; Tang et al., 2016; Young and McCoy, 2016; Friedrichsen and Engelmann, 2017; Lindenmeier et al., 2017; Peyer et al., 2017; Antonetti et al., 2018; Beldad and Hegner, 2018; Sama et al., 2018; Yoganathan et al., 2018; Herédia-Colaço et al., 2019
Mixed methods	2	Long and Murray, 2013; O'Connor et al., 2017
Theoretical: Other	2	Balineau and Dufeu, 2010; Ballet and Carimentrand, 2010
Theoretical: Systematic literature review	1	Samoggia and Riedel, 2018
Grand total	32	

Thirty two articles were published in the period 2010–2018 (Figure 2). They described the mechanism behind purchasing fairly traded products. The number of articles has increased in the last 4 years in comparison to the 3 years before that.

In terms of outlets for these articles (Table 3), most (10) were published in the Journal of Business Ethics, while Food Quality and Preference ranked second with six articles. All other outlets published between two and one article in this period.

**Table 3:** Number of articles per outlet.

**Publication outlet**	**Number of articles**	**%**
Journal of Business Ethics	10	31%
Food Quality and Preference	6	19%
Journal of Business Research	3	9%
Appetite	2	6%
Ecological Economics	2	6%
Journal of Agricultural and Environmental Ethics	2	6%
Others	7	22%
Total	32	100%

The empirical research in these papers took place mainly in the countries specified in Table 4. This table shows that although fair trade is not established to the same degree in the USA as in Western Europe, nevertheless 22 (56%) samples in the empirical research are from European countries and 30% of the samples are from USA. This may also be related to the fact that some researchers have used online panels, such as MTurk, whose panels often sit in the United States.

**Table 4:** Location of empirical research.

**Country**	**Number of articles**	**%**
USA	11	28
Germany	4	10
UK	4	10
Netherlands	3	8
Belgium	2	5
Italy	2	5
Canada	2	5
Others	11	28
Total	39*^a^*	100

a *The total number refers to the different locations where empirical research has been taken place for the 32 articles presented in this review*.

## Thematic Review

Further to the thematic analysis of the documents, all relevant factors that contribute to the decision-making process behind purchases of fair trade products have been identified. Through a preliminary content analysis, we classified the articles into the following thematic areas: values, guilt and pride, willingness to pay, labels and certification, Theory of Planned Behavior and Theory of Reasoned Action, dual attitudes, religion and demographics. Then, a network analysis in Atlas.ti was carried out. The complete figure can be seen in Appendix A. Moreover, importantly, we used Atlas in order to identify relationships among the codes, as appropriate. Atlas allowed this hermeneutic approach to be conducted efficiently and with a great visual output that allows for quick understanding of the relationships with just one quick look at the networks. In most cases, there is no overlap of items in the different papers, as authors have examined the issue from different points of view. Therefore, in most cases the interrelated codes are within the same contribution.

### Values

Values can be viewed as desired end-states and influence the way people make decisions and act upon them. In that sense, they are highly relevant for decision-making processes for FT products.

In seven of the 32 articles values as put forward by Rokeach (1973) and Schwartz and Bilsky (1987) were used as a theoretical background. In his seminal research, Rokeach identified a list of universally relevant values, while Schwartz and Bilsky added structure to those values and constructed a theory of the “universal type of values by viewing values as cognitive representations of three universal requirements: (a) biological needs, (b) interactional requirements for interpersonal coordination, and (c) societal demands for group welfare and survival” (Schwartz and Bilsky, 1987, p. 550).

**Figure 3:**
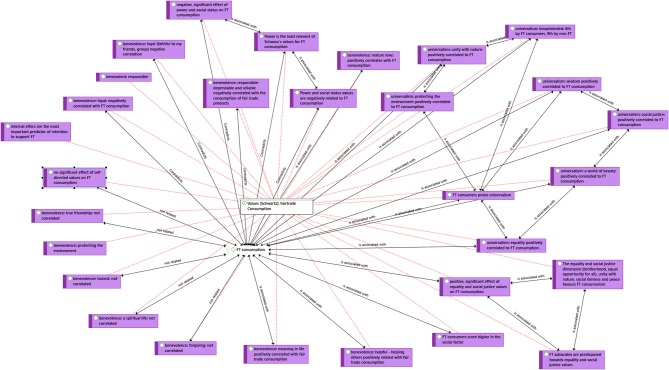
Network analysis: Values.

Based on Rokeach and Schwartz's theories and extended to include those values that are pertinent to ethical purchases (Ethical Consumption Values, ECV) is the core of Kim et al. article 2010. In this piece of research loyalty toward fair trade products was investigated through establishing the link between ECV and FT product beliefs and fair trade corporate evaluation. Bratanova et al. (2015) concluded that people rate food of ethical original as tastier than conventional food and validated this through four studies, proving the role of values in the decision-making. In another study universalism and benevolence values were investigated, concluding that an “overriding sense of responsibility to one's own group (in group) may override empathy with remote fair trade producers (out group)” (Doran, 2010). An adapted list of Rokeach values was tested with three classes of values being identified: self-directed—with no effect on fair trade consumption, equality and social justice—with positive effect, and power and social status—with negative effect (Ladhari and Tchetgna, 2015). Chatzidakis et al. (2016) conclude that internal ethics is the most important predictor of intention, while he proposes internal ethics are linked to a person's identity which is a product of his moral values. Peyer et al. (2017) conclude that voluntary simplifiers tend to have more universalistic values and to buy more green products, including FT products. Long and Murray (2013) use convergence and divergence of values. In this piece of research consumers purchasing several ethical goods experience convergence, in that they believe that these products support similar values. Divergence on the other hand is when they believe, that purchasing one product supports values which are not in accordance with the values of the other products. In their research they identified two groups: local globalists, for whom local food purchases and FT food purchases from overseas do not come to a conflict of values and the food patriots for whom it is important to support only the local producers. Surprisingly Ballet and Carimentrand (2010) predict that due to the distance created the shopper and the farmer due to the new FT structures which departed from the “third world shop” paradigm and moved into modern trade will cause a depersonalization of ethics that may be harmful for FT. Although this opinion has merit, the contrary has been in practice the case with FT sales booming since 2010. We present the above relationships in Figure 3. We highlight the factors that are not related to consumption of FT products but have nonetheless been studied in the literature. Moreover, we have highlighted the factors that have a negative effect (contradicts). With “is associated with” we have flagged all factors that have either a positive effect or are interrelated.

### Guilt and Pride

**Figure 4:**
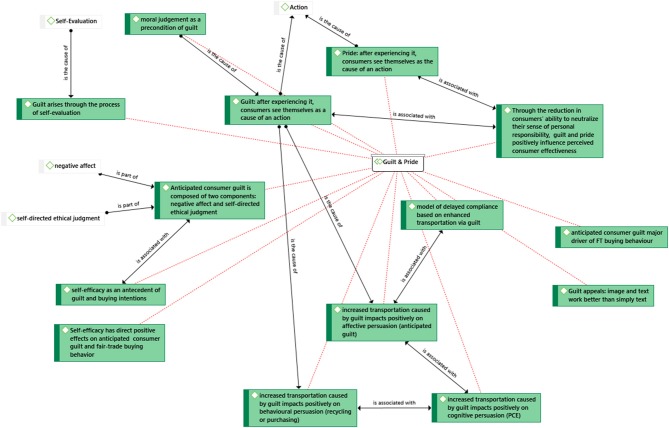
Network analysis: Guilt and pride.

Guilt is the focal point of three articles, while pride features in one of those articles. Guilt is a significant factor in the consumer decision-making process, as increased transportation caused by guilt impacts positively on affective, cognitive and behavioral levels, even if there is a temporal delay between message and act of compliance (Antonetti et al., 2018). After experiencing guilt and pride consumers see themselves as the cause of an action (Antonetti and Maklan, 2014). Consumers overcome neutralization and have an increased sense of perceived consumer effectiveness (Antonetti and Maklan, 2014). Anticipated consumer guilt arises within the process of self-realization and was found to mediate the effects of its antecedents on FT buying intention with FT consumers scoring higher in the social factor. Anticipated consumer guilt consists of two components: “negative affect and self-directed ethical judgment” (Lindenmeier et al., 2017). Finally, self-efficacy was proven to have “direct positive effects on anticipated consumer guilt and fair-trade buying behavior” (Lindenmeier et al., 2017, p. 9). We have identified the above relationships in a network in Figure 4. We have identified causalities under “is the cause of,” associations “is associated with,” or components “is part of.”

### Willingness to Pay

Willingness to pay was mentioned in nine of the articles in the final list. Multisensory marketing techniques can increase willingness to pay (Yoganathan et al., 2018). While consumers who buy FT coffee are willing to pay a considerable premium, the size of it varies per country (Samoggia and Riedel, 2018). In Belgium consumers are only willing to pay 10% extra for FT coffee (Samoggia and Riedel, 2018). Moral satisfaction also leads to a higher WTP through enhanced taste expectations (Bratanova et al., 2015). Voluntarism moderates the WTP for fair trade chocolate, while citizenship moderates the WTP for Fair Trade and Carbon Footprint products (Young and McCoy, 2016). Higher WTP for FT chocolate as for Rainforest or carbon footprint may be related to the expressed concern of participants for working conditions and human rights (Vecchio and Annunziata, 2015). For food choices consumers who spend more time and fixate more on sustainability attributes have a higher preference for these attributes and WTP (Van Loo et al., 2015). WTP for the attributes “organic, FT and donations via Cause-related Marketing (CrM) vary significantly among the group studied” (Langen, 2011).

### Labels and Certification

**Figure 5:**
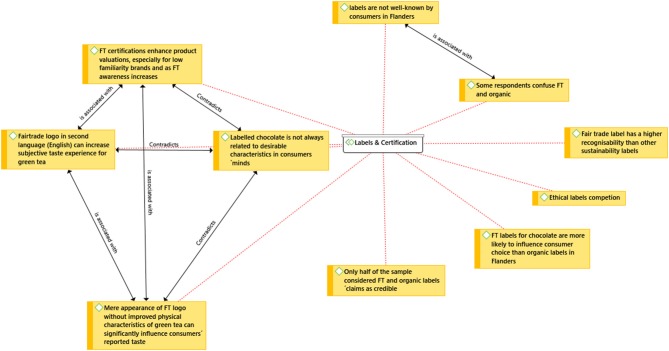
Network analysis: Labels and certification.

Fair trade certifications enhance product valuations, especially for low familiar brands, among consumers with increased expertise (Herédia-Colaço et al., 2019). The mere appearance of the FT logo improves the taste perception of green tea (Tang et al., 2016). Although label credibility can be low, the label has a higher recognisability than other sustainability labels (Rousseau, 2015). Cultural factors may influence the level of expertise. In Herédia-Colaço et al. (2019) research “[e]specially in more (mature) individualistic markets (vs. collectivistic) consumer ethical behavior seems to be greatly influenced by consumers' perceptions about the eligibility of brands using (or not) fair trade. This effect is strengthened by the significant mediating role of consumers' ethicality perceptions on the relationship between fair trade and the willingness to pay for brands” (Herédia-Colaço et al., 2019). Consumers are willing to pay a price premium for the three ethical food attributes organic, FT and low carbon emissions. Generally these labels are not in competition, unless “(1) the price of organic foods is decreased significantly, (2) the price for fair trade food products is set higher than consumers' WTP, and (3) bananas labeled as having lower carbon footprint are made available in retail stores and sold at a price lower than consumers' WTP” (Akaichi et al., 2016). In a study conducted in Flanders, Belgium, fair trade labels for chocolate were more likely to influence consumer choice for chocolate, than organic labels. The organic label seems superfluous for most consumers as “[l] labeled chocolate is not always related to desirable characteristics in consumers' minds” (Rousseau, 2015, p. 98). Tea with the FT logo was also found to taste better, while its appearance in a second language improved this experience (Tang et al., 2016). The above observations have also been captured in Figure 5 below.

### Theory of Planned Behavior and Theory of Reasoned Action

**Figure 6:**
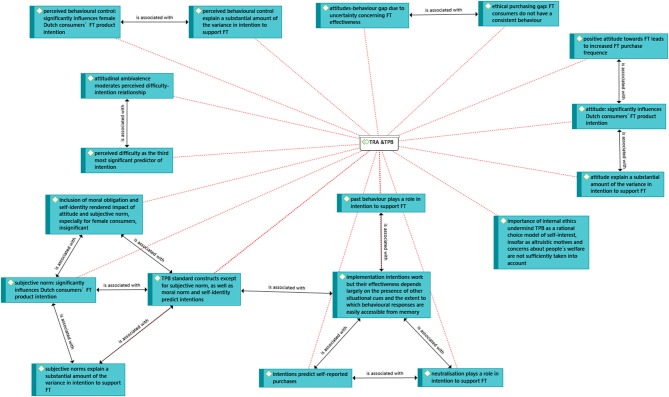
Network analysis: TRA and TPB.

The Theory of Planned Behavior (TPB) (Ajzen, 1991) and Theory of Reasoned Action (TRA) (Fishbein and Ajzen, 1975) have been used by numerous academics as a theoretical framework for the understanding of purchase decisions for ethical goods and sustainable consumption more generally (Ramayah et al., 2010; Paul et al., 2016; Wiederhold and Martinez, 2018; for example Minton et al., 2018). Specifically in the framework of this paper, five papers have been identified. Balineau and Dufeu (2010) in their theoretical paper present fair trade goods as credence goods, but rather with the full definition than with the usual classification. They find that FT goods can be at best described as indeterminate goods as proposed by Lupton (2005). The indeterminacy of FT products, more specifically the fact that consumers do not understand their effectiveness, may be another factor which contributes to the attitudes-behavior gap. In a empirical study, TPB was tested in the Netherlands revealing that “attitude, subjective norm, and perceived behavioral control significantly influence those consumers' FT product purchase intention (except for male consumers in which perceived behavioral control has no effect at all)”(Beldad and Hegner, 2018). In the same study, an extended version of the TPB was tested, including moral obligation and self-identity. These rendered impact of attitude and subjective norm, especially for female consumers, insignificant. In Chatzidakis et al. research 2016 an integrated model including measures of the theory of planned behavior, personal norms, self-identity, neutralization, past experience, and attitudinal ambivalence is proposed. The paper concludes that the “measure of ‘internal ethics' was the most important predictor of intention over and above traditional determinants such as attitude and subjective norms” (Chatzidakis et al., 2016). Therefore, rational considerations are less important than such subjective measures. In another piece of research (Fennis et al., 2011) implementation intentions are identified to work but their effectiveness depends largely on the presence of other situational cues and the extent to which behavioral responses are easily accessible from memory. O'Connor et al. (2017) conclude that TPB standard constructs except for subjective norm, as well as moral norm and self-identity predict intentions which, in turn, predict fair trade purchasing behavior. The following TPB beliefs of “making me feel good”, “reflecting my values”, and “being unable to afford Fair Trade products” can be used to identify buyers from non-buyers of FT products and as such can be useful cues for FT campaigners and marketers (O'Connor et al., 2017). We identified all of the above relations in the network in Figure 6. Moreover, we identified and highlighted relations among the items under “is associated with.”

### Other Themes

The following themes have also been identified, albeit in a small number of articles each.

#### Dual Attitudes

Although the theory of dual attitudes can offer very interesting insights into such “consumption gaps” as the one relating to FT products, we only identified one paper that has explicitly drawn on this theory in our systematic literature review. According to the notion of dual attitudes, explicit attitudes are those that individuals claim, especially under the influence of social desirability bias, whereas implicit attitudes are the ones that actually guide their behavior (Greenwald and Banaji, 1995). Govind et al. (2019) proved through two longitudinal studies in their research that dual attitudes exist and as such offer an interesting field for further research for FT products.

#### Religion

As far as religion is concerned, non-religious people are more likely to intend to buy FT products, while from the religious groups Buddhist have a higher probability to purchase FT products than Catholics or Protestants (Doran and Natale, 2011).

#### Demographics

In line with research published on ethical consumerism, demographics lead to confusing results or deem to be irrelevant (Langen, 2011).

## Discussion

Due to the sharply increasing consumption levels in many parts of the world, humanity is facing with immense issues that need to be tackled in order to guarantee a bright future for generations to come. We argue that consumption needs to depart from existing, traditional patterns, as these are disastrous not only for the environment but also for the weakest on the supply chain, which are often those who produce the food we eat and the clothes we wear. FT aims to support farmers and workers to earn a fair pay for their efforts, but also help communities invest in infrastructure, so that the future of next generations is guaranteed, through for example better roads and sanitation, health services and so on. Moreover, through FT farmers receive support in the form of schooling and interest-free loans in order to improve their agricultural practices (Fairtrade Labelling Organizations International (FLO), 2018). At the same time, it seems that the fair trade certification can be a win-win situation for businesses too, as through higher investment in infrastructure and mitigation of risk through enhanced agricultural practices, the occurrence of supply chain shocks, which are becoming more often due to changing weather patterns, can be minimized.

## Summary of Evidence and Practical Implications

Consumers find that morality “tastes” good (Bratanova et al., 2015; Tang et al., 2016) and that ethical products are less calorific (Schuldt et al., 2012). This increases the WTP for these products. The FT label has a higher recognisability than other labels (Rousseau, 2015). Consumers who are interested in sustainability characteristics tend to fixate longer on such products, which suggests that a prominent placement of the FT certification mark can increase fixation time on those products (Van Loo et al., 2015) and as such increase the probability that these are purchased and not the competitive products. As consumers find that FT products taste good (Bratanova et al., 2015; Tang et al., 2016) the costs related to the license of the FT mark should be less of an obstacle for brands considering whether they decide for FT certification or not.

Research indicated that WTP varies per country studied (Kim et al., 2010; Herédia-Colaço et al., 2019). Therefore, marketers should conduct local research in order to identify the exact values. Importantly FT certifications enhance product valuation, despite brand and cultural influences. However, consumers have specific perceptions as to which brands are eligible to be FT certified (Herédia-Colaço et al., 2019). Therefore, a marketer considering using the FT certification should test its acceptance among its loyal consumers. Multisensory techniques may increase WTP for FT products when purchasing online (Yoganathan et al., 2018), which suggests that using new, immersive technologies can be an effective marketing tool to attract more consumer interest for FT products.

Social desirability bias, the motivation to consume ethically in front of others, or to claim to consume ethically more than one actually does, has been proven to be a key theme in the consumer decision-making process (Kimura et al., 2012; Chatzidakis et al., 2016; Friedrichsen and Engelmann, 2017). Subjective norm has also been identified to play a significant role in the decision-making, reinforcing the above point (Chatzidakis et al., 2016; Beldad and Hegner, 2018). To derive managerial implications from this insight further research is necessary.

As an extension to the above point, another area which merits more academic research is dual attitudes. Govind et al. (2019) confirm the existence of different implicit and explicit motivations (attitudes) in the purchase of fair trade goods. Strikingly “[e]ven though explicit attitudes react to the stimuli presented, our findings suggest they have no impact on the choice of consumers” (Govind et al., 2019). As this is the only paper to the best of our knowledge on dual attitudes in the context of fair trade products, further research is necessary to derive sound managerial implications and offer tangible insights to the marketing professionals.

Demographic factors do not appear to play a significant role in the decision-making process, in line with previously published research. There is no general consensus in academic literature as to the demographics of the ethical consumer. In the literature review conducted by Bray et al. (2011) the findings are “conflicting and confusing”; older consumers and women are ethically more sensitive. Ethical sensitivity also rises with affluence and is greater at lower educational levels. However, in Doran's (2009, p. 558) research age, gender, race, education or marital status were proven to be poor predictors for Fair Trade consumption. Bray et al. (2011) add that various authors find no such correlations and therefore demographic factors are poor predictors of ethical behavior. As a result, FT marketers should not attempt to focus their efforts on a specific age group or other group based purely on demographic characteristics.

Research has identified that specific values, such as universalism or broadmindedness (Doran, 2010; Ladhari and Tchetgna, 2015) can increase preference for fair trade products. Market segmentation is an indispensable part of marketing and commercial strategies to identify target groups and develop accordingly appropriate commercial and communication strategies. As such, values should be considered in market segmentation attempts.

Guilt impacts positively on affective, cognitive and behavioral levels, even if there is a temporal delay between message and act of compliance (Antonetti et al., 2018). Moreover, both after experiencing guilt and pride consumers see themselves as the cause of an action and become motivated to purchase FT products (Antonetti and Maklan, 2014). Further research is here required to identify specific messaging strategies and pin-point the right tonality in the communication lines of the producers of FT products and fair trade as such to consumers.

### Limitations

Despite thorough research in three leading databases, the article does not include publications outside those, as well as non-English publications. This may lead to an omission of culturally specific papers. Cultural differences have been identified in the analysis, therefore inclusion of publications in other languages would of academic interest. All publications included in this review are double-blind-reviewed journal articles, therefore contributions in other forms have been excluded from this review. This paper is a systematic review, therefore no attempts have been made to compare and contrast the impact of different factors on the decision-making for fairly traded products, but rather to present the multitude of factors that contribute to this phenomenon. A meta-analysis synthesizes information obtained as a result of several studies on one topic (Jacoby and Ciuk, 2017) or one research question. A meta-analysis would thus be not only an interesting, but also a meaningful next step. This would require a relatively narrow formulation of the research question, so that the research outcomes of the different publications offer a relatively the necessary degree of comparability. Moreover, a thorough examination of risk of bias in each of the studies presented here would be recommendable. The focus of this review limits itself to fairly traded products, although there is a large number of contributions for areas such as green consumerism, ethical consumerism and conscientious consumerism. A comparative analysis would also be interesting in order to identify factors, as well as tools and methods employed in this broader field of research. Their applicability could then be considered for fair-trade consumerism and help guide future research.

## Conclusion

Through this systematic review and the resulting analysis in Atlas.ti, we identified factors that moderate FT consumption and influence the decision-making behind it. A promising field for further research proves to be the area of dual attitudes, which to the best of our knowledge has only been tested once for FT products. The theory of dual attitudes has been successfully tested for other types of virtuous or non-virtuous consumption. Although consumers find that “moral” tastes better, the self-claimed FT consumption still lags behind actual consumption. Most interestingly FT coffee, which in the most established FT markets has been in sale for almost 25 years and can be found in almost all stores, still enjoys very low market shares. For FT coffee a further analysis of implicit attitudes, as well as suitable nudges to increase its consumption would be of immense interest not only for academics, but also for practitioners and due to its social impact for society as a whole.

## Data Availability Statement

The datasets generated for this study are available on request to the corresponding author.

## Author Contributions

EK and MG-S contributed to the conceptualization, design, to the drafting and revising of the manuscript, analyzed the data to ensure objectivity in a double blind-review, and are responsible for the final version of the manuscript and take equal responsibility for results published. EK conducted the literature search, the search, data selection and extraction between the 13th of November and 16th of December 2018, and coded the resulting papers. MG-S crossed-validated the data selection and data extraction.

### Conflict of Interest

The authors declare that the research was conducted in the absence of any commercial or financial relationships that could be construed as a potential conflict of interest.
