# Supplementary material for: Words-Deeds Gap for the Purchase of Fairtrade Products: A Systematic Literature Review
Source: Front Psychol. 2019 Dec 3;10:2705. doi: 10.3389/fpsyg.2019.02705 (PMC6902643; doi:10.3389/fpsyg.2019.02705)
Supplement: Supplementary file 1 [file Data_Sheet_1.docx]

## Appendix A

### Network Analysis


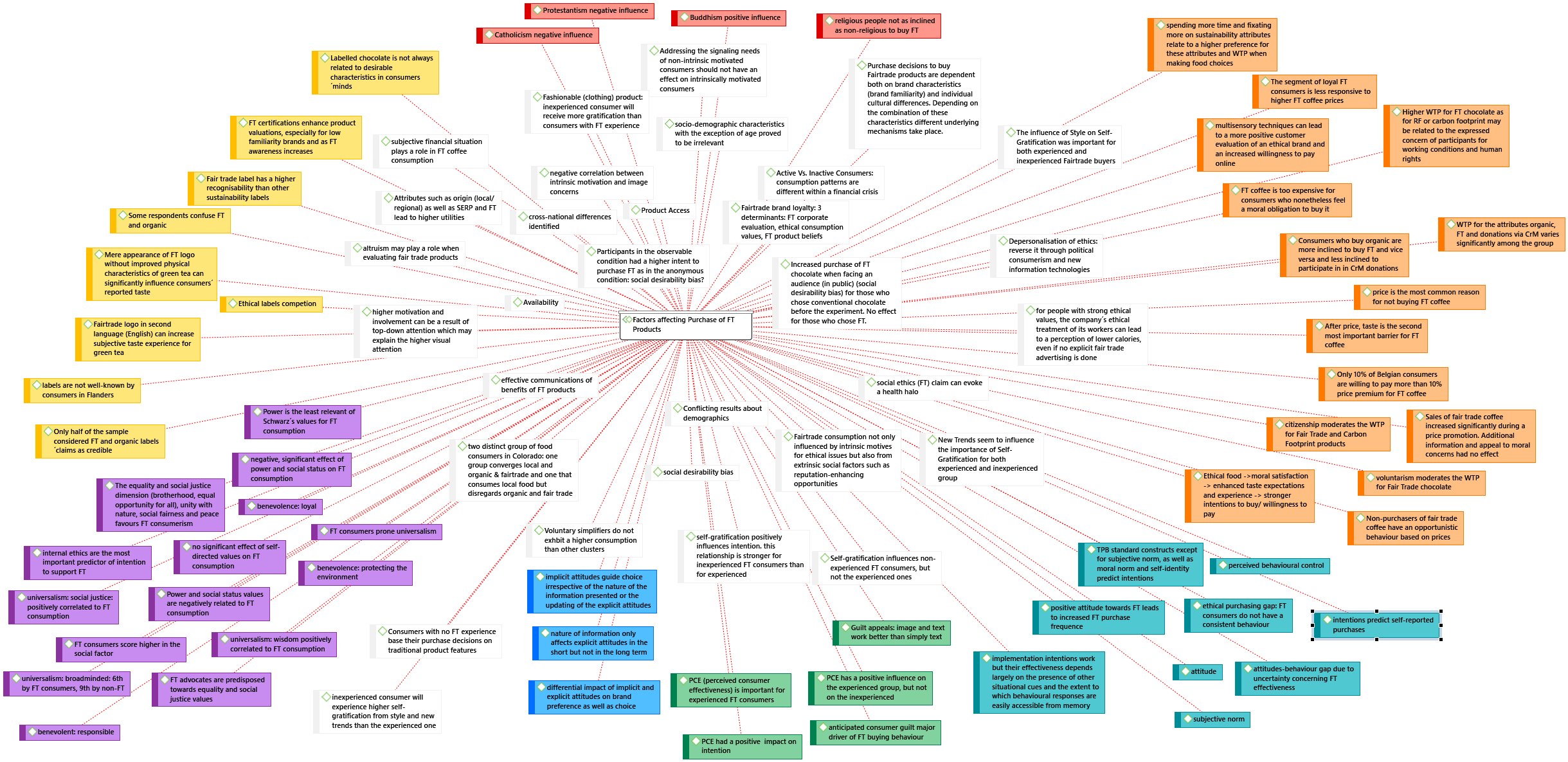


Supplementary Figure 1 Network Analysis: Factors Affecting Purchase of FT products

The different colours signify the different **thematic areas** of the codes. Purple-coloured codes refer to those codes who have a reference to Schwartz´s Values, coloured blue are those related to dual attitudes, orange those related to price or willingness to pay, while red refer to religious factors. We marked factors relating to labels and certification yellow, while turquoise marked factors relating to the Theory of Reasoned Action (TRA) or Theory of Planned Behaviour (TPB). Finally, green are those factors relating to guilt. All other factors which did not belong to a wider theoretical or thematic framework were intentionally left white. For a detailed presentation of these factors please view Table 5.

## Appendix B

## Taxonomy of Articles

| Authors | Document Title | Product | Research Design (Conclusive: Causal, Conclusive: Descriptive, Exploratory) | Research Method (Survey, Observation, Depth Interview, Focus Group, Preexperimental Design | Sampling Method | Sample Size | Factors |
| --- | --- | --- | --- | --- | --- | --- | --- |
| Akaichi, Faical; de Grauw, Steven; Darmon, Paul; Revoredo-Giha, Cesar | D 1: Does Fair Trade Compete with Carbon Footprint and Organic Attributes in the Eyes of Consumers? Results from a Pilot Study in Scotland, The Netherlands and France | bananas | Conclusive: Causal Research | True Experimental Designs | Probability: simple random | 100, 52, 95 | Ethical labels (FT, organic, lower carbon) competing not in current market situation, but only when 1. Price of organic food is significantly lower, 2. price of FT bananas is higher than WTP, 3. Lower carbon bananas are offered lower than WTP |
| Antonetti, Paolo; Baines, Paul; Jain, Shailendra | D 2: The persuasiveness of guilt appeals over time: Pathways to delayed compliance | no product | Conclusive: Descriptive, Exploratory | Survey, True Experimental Designs | Non-probability: snowball | 401 | Increased transportation caused by guilt impacts positively on affective, cognitive, and behavioural levels, even if there is a temporal delay between message and act of compliance |
| Antonetti, Paolo; Maklan, Stan | D 3: Feelings that Make a Difference: How Guilt and Pride Convince Consumers of the Effectiveness of Sustainable Consumption Choices | coffee | Exploratory | True Experimental Designs | Non-probability: convenience | 415 | Guilt and Pride: after experiencing one of those feelings, consumers see themselves as the cause of an action. Consumers overcome neutralisation and have an increased sense of perceived consumer effectiveness |
| Balineau, Gaëlle; Dufeu, Ivan | D 4: Are Fair Trade goods credence goods? A new proposal, with French illustrations | no product | Theoretical: Other |  |  |  | Attitudes-behaviour gap due to uncertainty concerning FT effectiveness; effective communications of benefits of FT products |
| Ballet, Jérôme; Carimentrand, Aurélie | D 5: Fair Trade and the Depersonalization of Ethics | no product | Theoretical: Other |  |  |  | Depersonalisation of ethics. Sense of enterprise: consumers see themselves as part of the production process: expansive ethics of care, relational ethics overcoming geographical, psychological, or cultural differences. Reduce the distance between producer and consumer through political consumerism and new technologies |
| Beldad, A.; Hegner, S. | D 6: Determinants of Fair Trade Product Purchase Intention of Dutch Consumers According to the Extended Theory of Planned Behaviour: The Moderating Role of Gender | no product | Conclusive: Causal Research | Survey | Probability: stratified: proportionate | 499 | Attitude, subjective norm, and perceived behavioural control significantly influence consumers’ FT product purchase intention (except for male consumers in which perceived behavioural control has no effect at all). However, the inclusion of moral obligation and self-identity rendered the impact of attitude and subjective norm (specifically, for female consumers) insignificant |
| Bondy, Tierney; Talwar, Vishal | D 7: Through Thick and Thin: How Fair Trade Consumers Have Reacted to the Global Economic Recession | food | Conclusive: Causal Research | Survey | Probability: simple random | 306 | Active Vs. Inactive FT Consumers: consumption patterns are different within a financial crisis. Inactive consumers will consume less, while active, except for the US Americans in the sample, will remain at the same levels. |
| Bratanova, Boyka; Vauclair, Christin Melanie; Kervyn, Nicolas; Schumann, Sandy; Wood, Robert; Klein, Olivier | D 8: Savouring morality. Moral satisfaction renders food of ethical origin subjectively tastier | apple juice, biscuits, chocolate | Conclusive: Causal Research | Survey, True Experimental Designs | Non-probability: convenience (2), Probability: simple random | 112, 4161, 50 | Ethical food ->moral satisfaction -> enhanced taste expectations and experience -> stronger intentions to buy/ willingness to pay |
| Chatzidakis, Andreas; Kastanakis, Minas; Stathopoulou, Anastasia | D 9: Socio-Cognitive Determinants of Consumers’ Support for the Fair Trade Movement | no product | Conclusive: Descriptive | Survey | Probability: simple random | 517 | Attitude, subjective norms, and perceived behavioural control explain a substantial amount of the variance in intention to support FT; internal ethics the most important predictor of intention: suggest that TPB as a model of rational, choice behaviour is limited in its explanatory power, neutralisation and past behaviour are also significant. |
| Doran, Caroline Josephine | D 10: Fair trade consumption: In support of the out-group | no product | Conclusive: Descriptive | Survey | Probability: simple random | 475, 809 | Positive influence on FT consumption: universalism values: broadminded, wisdom, social justice, equality, world at peace, world of beauty, unity with nature, protecting the environment. Benevolence values: helpful, mature love, meaning in life. Negative correlation: benevolence: honest, forgiving, true friendship, spiritual life. Neutral: benevolence values: loyal, responsible. |
| Doran, Caroline Josephine; Natale, Samuel Michael | D 11: (Empatheia) and Caritas: The Role of Religion in Fair Trade Consumption | no product | Conclusive: Descriptive | Survey, True Experimental Designs | Probability: simple random | 582 | Buddhism positive influence, Catholicism negative influence, Protestantism negative influence, religious people not as inclined as non-religious to buy FT |
| Fennis, Bob M.; Adriaanse, Marieke A.; Stroebe, Wolfgang; Pol, Bert | D 12: Bridging the intention–behavior gap: Inducing implementation intentions through persuasive appeals | no product | Conclusive: Causal Research |  | Non-probability: convenience | 217 | Implementation intentions work, but their effectiveness depends largely on the presence of other situational cues and the extent to which behavioural responses are easily accessible from memory |
| Friedrichsen, Jana; Engelmann, Dirk | D 13: Who Cares About Social Image? | chocolate | Conclusive: Causal Research | True Experimental Designs |  | 121, 144 | Increased purchase of FT chocolate when facing an audience (in public) (social desirability bias) for those who chose conventional chocolate before the experiment. No effect for those who chose FT. intrinsic preferences and the concern for social approval are negatively correlated. negative correlation between intrinsic motivation and image concerns. |
| Govind, Rahul; Singh, Jatinder Jit; Garg, Nitika; D'Silva, Shachi | D 14: Not Walking the Walk: How Dual Attitudes Influence Behavioral Outcomes in Ethical Consumption | chocolate, coca cola, coffee, ice cream, soft drinks | Conclusive: Descriptive | Survey, True Experimental Designs | Non-probability: convenience | 50, 78 | Differential impact of implicit and explicit attitudes on brand preference as well as choice; implicit attitudes guide choice irrespective of the nature of the information presented or the updating of the explicit attitudes; nature of information only affects explicit attitudes in the short but not in the long term; social desirability bias |
| Herédia-Colaço; Vera Coelho do Vale, Rita; Villas-Boas, Sofia B. | D 15: Does Fair Trade Breed Contempt? A Cross-Country Examination on the Moderating Role of Brand Familiarity and Consumer Expertise on Product Evaluation | chocolate, detergent, rice, tea, tissues | Conclusive: Causal Research | Survey, True Experimental Designs | Non-probability: convenience | 103, 315, 508 | Fair trade certifications enhance product valuations; Purchase decisions to buy Fairtrade products are dependent both on brand characteristics (brand familiarity) and individual cultural differences. Depending on the combination of these characteristics different underlying mechanisms take place; Cultural differences may account for some of the unexplained variation in choice behaviours observed across countries, especially in more (mature) individualistic markets (vs. collectivistic) consumers´ ethical behaviour seems to be greatly influences by consumers´ perceptions about the eligibility of brands using or not FT. This effect is strengthened by the significant mediating role of consumers´ ethicality perceptions on the relationship between FT and WTP |
| Kim, Gwang-Suk Suk; Lee, Grace Y.; Park, Kiwan | D 16: A Cross-National Investigation on How Ethical Consumers Build Loyalty Toward Fair Trade Brands | coffee, toilet paper | Conclusive: Descriptive | Survey |  |  | Cross-national differences identified; Fairtrade brand loyalty: 3 determinants: FT corporate evaluation, ethical consumption values, FT product beliefs |
| Kimura, Atsushi; Mukawa, Naoki; Yamamoto, Mana; Masuda, Tomohiro; Yuasa, Masahide; ichi Goto, Sho; Oka, Takashi; Wada, Yuji | D 17: The influence of reputational concerns on purchase intention of fair-trade foods among young Japanese adults | chocolate | Conclusive: Causal Research | Survey, True Experimental Designs | Non-probability: convenience | 106, 84 | Fairtrade consumption not only influenced by intrinsic motives for ethical issues but also from extrinsic social factors such as reputation-enhancing opportunities; Participants in the observable condition had a higher intent to purchase FT as in the anonymous condition. |
| Ladhari, Riadh; Tchetgna, Nina Michelè | D 18: The influence of personal values on Fair Trade consumption |  | Conclusive: Descriptive | Survey | Non-probability: convenience | 268 | FT advocates are predisposed towards equality and social justice values; FT consumers prone universalism; negative, significant effect of power and social status on FT consumption; no significant effect of self-directed values on FT consumption; positive, significant effect of equality and social justice values on FT consumption; Power and social status values are negatively related to FT consumption; Power is the least relevant of Schwarz´s values for FT consumption; The equality and social justice dimension (brotherhood, equal opportunity for all), unity with nature, social fairness and peace favours FT consumerism |
| Langen, Nina | D 19: Are ethical consumption and charitable giving substitutes or not? Insights into consumers' coffee choice | coffee | Conclusive: Causal Research | Depth Interviews, True Experimental Designs | Non-probability: convenience | 484 | For 27% of the 484 respondents, ethical consumption occurs at the expense of other forms of ethical behaviour such as charitable giving. Consumers who buy organic are more inclined to buy FT and vice versa and less inclined to participate in in CrM donations; socio-demographic characteristics, apart from age proved to be irrelevant; WTP for the attributes organic, FT and donations via CrM varies significantly among the group |
| Lee, Min Young; Jackson, Vanessa; Miller-Spillman, Kimberly A.; Ferrell, Erika | D 20: Female consumers' intention to be involved in fair-trade product consumption in the U.S.: The role of previous experience, product features, and perceived benefits | clothing | Conclusive: Descriptive | Survey | Non-probability: snowball | 278 | Consumers with no FT experience base their purchase decisions on traditional product features (style, new trends) as in this way they experience higher self-gratification, than the experienced ones; Style and New Trends are antecedents to Self-Gratiﬁcation and PCE which inﬂuences the intention to participate in fair-trade product consumption regardless of previous experience. Fashionable product: inexperienced FT consumer receives higher gratiﬁcation than experienced FT consumer. New Trends seem to inﬂuence the importance of Self-Gratification for both experienced and inexperienced group; PCE (perceived consumer effectiveness) is important for experienced FT ; The inﬂuence of Style on Self-Gratiﬁcation was important for both experienced and inexperienced Fairtrade consumers; PCE had a positive impact on intention; PCE has a positive influence on the experienced group, but not on the inexperienced; Self-gratification influences non-experienced FT consumers, but not the experienced ones; self-gratification positively influences intention. this relationship is stronger for inexperienced FT consumers than for experienced |
| Lindenmeier, J.; Lwin, M.; Andersch, H.; Phau, I.; Seemann, A.-K. | D 21: Anticipated Consumer Guilt: An Investigation into its Antecedents and Consequences for Fair-Trade Consumption | clothing | Conclusive: Descriptive | Survey | N/A | 430 | Anticipated consumer guilt mediates the effects of its antecedents on FT buying intention; FT consumers score higher in the social factor; Anticipated consumer guilt is composed of two components: negative affect and self-directed ethical judgment; self-efficacy as an antecedent of guilt and buying intentions |
| Long, Michael a.; Murray, Douglas L. | D 22: Ethical Consumption, Values Convergence/Divergence and Community Development | food | Conclusive: Descriptive | Focus Groups Interviews, Survey | Probability: simple random | 23, 469 | Two distinct group of food consumers in Colorado: one group converges local and organic & FT and one that consumes local food but disregards organic and fair trade |
| O'Connor, Erin L.; Sims, Lauren; White, Katherine M. | D 23: Ethical food choices: Examining people's Fair Trade purchasing decisions | no product | Conclusive: Descriptive, Exploratory | Focus Groups Interviews, Survey | Non-probability: convenience | 11, 178 | Intentions predict self-reported purchases; TPB standard constructs except for subjective norm, as well as moral norm and self-identity predict intentions |
| Peyer, Mathias; Balderjahn, Ingo; Seegebarth, Barbara; Klemm, Alexandra | D 24: The role of sustainability in profiling voluntary simplifiers |  | Conclusive: Descriptive | Survey | Non-probability: quota, Probability: stratified | 1458 | Voluntary simplifiers do not exhibit a higher consumption than other clusters |
| Rousseau, Sandra | D 25: The role of organic and fair trade labels when choosing chocolate | chocolate | Conclusive: Causal Research, Conclusive: Descriptive | Survey, True Experimental Designs | Non-probability: convenience, Non-probability: snowball | 601 | Fair trade label has a higher recognisability than other sustainability labels; FT more likely to influence purchase as other sustainability labels; Labelled chocolate is not always related to desirable characteristics in consumers´ minds; labels are not well-known by consumers in Flanders; Only half of the sample considered FT and organic labels´ claims as credible; Some respondents confuse FT and organic |
| Sama, Celia Crespo-Cebada, Eva Díaz-Caro, Carlos Escribano, Miguel Mesías, Francisco J. | D 26: Consumer Preferences for Foodstuffs Produced in a Socio-environmentally Responsible Manner: A Threat to Fair Trade Producers? | honey | Conclusive: Descriptive | Survey | Probability: stratified: proportionate | 461 | Attributes such as origin (local/regional) as well as socio-environmentally responsible production (SERP) and FT lead to higher utilities than conventional products |
| Samoggia, Antonella; Riedel, Bettina | D 27: Coffee consumption and purchasing behavior review: Insights for further research | coffee | Theoretical: Systematic literature review |  |  |  | FT coffee is too expensive for consumers who nonetheless feel a moral obligation to buy it; After price, taste is the second most important barrier for FT coffee; availability; product access; conflicting results about demographics; ethical purchasing gap: FT consumers do not have a consistent behaviour; Non-purchasers of fair trade coﬀee have an opportunistic behaviour based on prices; Only 10% of Belgian consumers are willing to pay more than 10% price premium for FT coffee; positive attitude towards FT leads to increased FT purchase frequence; Product Access; Availability; Sales of fair trade coﬀee increased significantly during a price promotion. Additional information and appeal to moral concerns had no effect; subjective financial situation plays a role in FT coffee consumption; The segment of loyal FT consumers is less responsive to higher FT coffee prices |
| Schuldt, Jonathon P.; Muller, Dominique; Schwarz, Norbert | D 28: The "Fair Trade" Effect: Health Halos From Social Ethics Claims | chocolate | Conclusive: Causal Research, Conclusive: Descriptive | Survey, True Experimental Designs | Non-probability: convenience, Probability: simple random | 192, 56 | For people with strong ethical values, the company´s ethical treatment of its workers can lead to a perception of lower calories, even if no explicit fair trade advertising is done; social ethics (FT) claim can evoke a health halo |
| Tang, Shi; Arciniegas, Catalina; Yu, Feng; Han, Ji; Chen, Shuquan; Shi, Jinfang | D 29: Taste moral, taste good: The effects of Fairtrade logo and second language on product taste evaluation | tea | Conclusive: Causal Research | True Experimental Designs | Non-probability: convenience | 74 | Fairtrade logo in second language (English) can increase subjective taste experience for green tea; higher motivation and involvement can be a result of top-down attention which may explain the higher visual attention; Mere appearance of FT logo without improved physical characteristics of green tea can significantly influence consumers´ reported taste |
| Van Loo, Ellen J.; Caputo, Vincenzina; Nayga, Rodolfo M.; Seo, Han-Seok Seok; Zhang, Baoyue; Verbeke, Wim | D 30: Sustainability labels on coffee: Consumer preferences, willingness-to-pay and visual attention to attributes |  | Conclusive: Causal Research, Conclusive: Descriptive | Survey, True Experimental Designs | Probability: stratified: disproportionate | 81 | Spending more time and fixating more on sustainability attributes relate to a higher preference for these attributes and WTP when making food choices |
| Young, Michael E.; McCoy, Anthony W. | D 31: Willingness-to-pay for sustainability-labelled chocolate: An experimental auction approach | chocolate | Conclusive: Causal Research | True Experimental Designs | Non-probability: convenience | 80 | Altruism may play a role when evaluating fair trade products; citizenship moderates the WTP for FT and carbon footprint products, voluntarism moderates the WTP for Fair trade Chocolate; Higher WTP for FT chocolate as for Rainforest or carbon footprint may be related to the expressed concern of participants for working conditions and human rights; voluntarism moderates the WTP for Fair Trade chocolate |
| Yoganathan, Vignesh; Osburg, Victoria-Sophie; Akhtar, Pervaiz | D 32: Sensory stimulation for sensible consumption: Multisensory marketing for e-tailing of ethical brands |  | Conclusive: Causal Research | True Experimental Designs | Probability: simple random | 308 | Multisensory techniques can lead to a more positive customer evaluation of an ethical brand and an increased willingness to pay online |

Supplementary Table 1 Appendix. Taxonomy of Articles
